# The perceived catchiness of music affects the experience of groove

**DOI:** 10.1371/journal.pone.0303309

**Published:** 2024-05-15

**Authors:** Toni Amadeus Bechtold, Ben Curry, Maria Witek

**Affiliations:** 1 Department of Music, University of Birmingham, Birmingham, United Kingdom; 2 Lucerne School of Music, Lucerne University of Applied Sciences and Arts, Lucerne, Switzerland; ’Enrico Fermi’ Research Center, ITALY

## Abstract

Catchiness and groove are common phenomena when listening to popular music. Catchiness may be a potential factor for experiencing groove but quantitative evidence for such a relationship is missing. To examine whether and how catchiness influences a key component of groove–the pleasurable urge to move to music (PLUMM)–we conducted a listening experiment with 450 participants and 240 short popular music clips of drum patterns, bass lines or keys/guitar parts. We found four main results: (1) catchiness as measured in a recognition task was only weakly associated with participants’ perceived catchiness of music. We showed that perceived catchiness is multi-dimensional, subjective, and strongly associated with pleasure. (2) We found a sizeable positive relationship between PLUMM and perceived catchiness. (3) However, the relationship is complex, as further analysis showed that pleasure suppresses perceived catchiness’ effect on the urge to move. (4) We compared common factors that promote perceived catchiness and PLUMM and found that listener-related variables contributed similarly, while the effects of musical content diverged. Overall, our data suggests music perceived as catchy is likely to foster groove experiences.

## Introduction

Whether tapping along to a rock song playing on the radio, swaying your body with the beat of a soothing ballad, or just letting go on a dancefloor–feeling the urge to move to music is a familiar sensation to many people. This “pleasant sense of wanting to move along with the music” [1] or pleasurable urge to move to music (PLUMM [2, 3]) constitutes a central aspect of what music psychologists call groove.

In many studies, the groove experience has been equaled to PLUMM, but some accounts have shown a more nuanced picture [4, 5], added a social dimension [6], or described it as a state of being [7]. Duman et al. [8] developed a comprehensive definition: “Groove is a participatory experience (related to immersion, movement, positive affect, and social connection) resulting from the subtle interaction of specific music- (such as time- and pitch- related features), performance-, and/or individual-related factors” (p.19). In the present text, we use PLUMM when referring specifically to the pleasure and urge to move aspects of groove, and groove when the concept goes beyond PLUMM, such as the experience as a whole or research on groove in general. Recent comprehensive overviews on groove research can be found in Câmara & Danielsen [9] and Etani et al. [10].

Many studies have examined musical features that were hypothesized to afford or intensify PLUMM, such as microtiming [11–17], syncopation [17–26], bass frequencies [27–30], tempo [31, 32], or harmonic complexity [22]. Yet, some studies found that the listener is more important than the musical features: PLUMM is more likely when music is familiar [33, 34] or matches musical taste [27, 29, 33, 35]. The role of expertise is less clear [22, 25, 33], and research on effects of cultural background is scarce [36].

The “psychological model of groove” [37–39] synthesized these insights and formulated hypotheses of how four mental processes (pleasure, inner representation of temporal regularity, time-related interest, energetic arousal) foster the urge to move depending on the music, listener, and situation. This shift towards mental processes reflects the continuing lack of clarity regarding the role of musical features and the complexity added by listener-related factors.

Pleasure, interest, and energetic arousal are of course not limited to groove and form important reactions to music in their own right. In a similar vein, there are other reactions to music that might interact with or influence the experience of groove. A promising candidate is catchiness, itself said to be vital for popular music [40]. It often coincides with groove in popular music, e.g., a catchy melody over a driving beat or a riff that is equally groovy and catchy. However, empirical support for a relationship between groove and catchiness has been sparse and often merely insinuated. For example, in a recent study in which participants reported their own definitions of groove, ‘catchy’ came up several times [8].

Van Balen [40] summarized that catchiness is “difficult to define, but it is generally understood as ‘easily recalled to memory’, or ‘memorable’” (p. 183). Burgoyne et al. [41] defined it as “long-term musical salience, the degree to which a musical fragment remains memorable after a period of time” (p. 1). Saliency and memorability link catchiness with musical hooks [42, 43], to the point that hooks have been defined as catchy passages of music [44]. Similarly, earworms or involuntary musical imagery (INMI) are often related to catchy music [45–47]. The fact that movement to INMI is common [48, 49] and makes earworms more likely [50] further suggests a connection between catchiness and PLUMM.

Catchiness has been hypothesized to arise directly from musical features, especially melodies, in theoretical [42, 51–53] and empirical accounts [54–56]. It is generally assumed that listeners can discriminate between more and less catchy music [40]. When considered synonymous with memorability, catchiness is often measured with recognition tasks. Some studies have quantified catchiness as the elapsed time until participants recognized a song [41, 57], others have counted how many participants sung along during a performance [58]. For both approaches, familiarity with the music is a decisive precondition. In contrast, Russell [54] measured the memorability of unfamiliar music in a two-phased recognition experiment and quantified it as the percentage of correct recognitions. He found that music rated as familiar, pleasing, melodious, or of low complexity is easier to recognize. In contrast, later studies [40, 41] have focused on connecting catchiness to acoustic musical properties analysed through music information retrieval. To our knowledge, there has been no study that combines musical, listener-related, behavioral, and subjective factors to investigate musical catchiness.

Previously, we explored a possible relationship between groove and catchiness using in-depth expert interviews with music creators, examining how they conceptualize, relate, and employ the two concepts in theory and practice [59]. The results suggested that musicians think of groove as a bodily experience coupled with positive affect, participation, immersion, and social aspects. Catchiness was conceptualized beyond mere memorability and not innately inherent to music, but as a perceived musical quality that “depends on the listener’s perception and experience of music, in which memorization and positive affect are central, and engagement, immediacy and clarity are other aspects” (p. 353). We refer to this concept as perceived catchiness. Ontologically, groove and perceived catchiness share positive affect as a key component, and we hypothesized that it functions as a hinge in their relationship. Further, we found several points of overlap in what make groove and catchiness more likely, such as accessibility, affectiveness, attention, and participation (p. 362ff). We found evidence that even single instrument patterns can promote groove and catchiness at the same time.

Given these findings, it is likely that the listener-related PLUMM-inducing factors, such as familiarity and musical taste, will also positively influence catchiness. For some other factors, differences are more likely: several studies have shown that complexity has an inverted-U relationship with PLUMM [17, 21, 22], while Russell [54] found a positively linear relationship with catchiness (as memorability). Considering musical content, research on groove has often focused on drums [17, 25, 32, 33] or non-melodic [22] stimuli to emphasize rhythmic structures. In contrast, research on catchiness or INMI has focused on melody [55, 56].

In the present study, we employed quantitative methods to examine the qualitative findings and theories in Bechtold et al. [59]. More precisely, we investigated the relationship between PLUMM and catchiness with four hypotheses:

Multi-dimensionality hypothesis (H_1_): We investigated how perceived catchiness relates to memorability and positive affect and predicted a close positive relationship of perceived catchiness with both. This was a necessary pre-examination for the following hypotheses.

Positive relation hypothesis (H_2_): We looked at the general relationship between catchiness and PLUMM in popular music patterns. We hypothesized that increased catchiness is associated with increased pleasure and urge to move.

Pleasure as hinge hypothesis (H_3_): We explored how catchiness and PLUMM are causally related, hypothesizing that pleasure is the mediator via which catchiness influences the urge to move.

Promoting factors hypothesis (H_4_): We compared musical, listener-related, behavioral, and subjective factors associated with PLUMM and catchiness to assess overlapping and diverging influences. We expected higher PLUMM ratings for drum patterns compared to higher catchiness ratings for melodic patterns (H_4a_), divergent effects of complexity (H_4b_), and an overlap of listener-related factors (H_4c_).

## Methods and materials

### Participants

We recruited participants on MTurk until we reached 450 valid data sets. Of these, 279 indicated to be male, 168 female, 1 specified as other, and 2 preferred not to answer. Mean age was 38 (range 21 to 72, SD = 10.44). Participants lived in the United States (347), India (61), Brazil (20) and in 12 other countries (22). Overall, they indicated fairly average musical preferences and expertise. To shorten the experiment, we selected one item from each of the scales in the 45-item Gold-MSI [60]: music engagement (mean = 3.51, SD = 1.15), music perception (mean = 3.69, SD = 1.02, both on Likert scales from 1 to 5), and music training (274 have played an instrument, 169 of these have practiced regularly for 3 or more years). Additionally, participants self-assigned their expertise on a slider (1–101) from music listener to professional musician (mean = 34.45, SD = 32.5). We asked participants how much they enjoy dancing (mean = 3.74, SD = 1.13) and how often they want to dance (mean 3.51, SD = 1.29) on 5-point Likert scales. Participants indicated with 7-point Likert scales how much they like 13 different popular music styles (overall mean = 5.10, SD = 0.89).

### Measures and tasks

We measured PLUMM with an established questionnaire that assesses the Urge to Move to music and Pleasure with three items each on a 7-point Likert scale [61] (H_2-4_). The Pleasure scale includes other affective phenomena—good mood and liking. Hence, it covers a broad part of the positive affect discussed in Bechtold et al. [59]. In previous studies, these two scales were strongly correlated [61, 62]. We did not measure groove in a more comprehensive understanding.

We measured catchiness in two different ways that each represent a different concept: Perceived Catchiness and catchiness as memorability, i.e. Recognition. A comparison of these two measures allowed to draw conclusions about how they relate and the multi-dimensionality hypothesis H_1_.

As there was no established questionnaire for measuring Perceived Catchiness, we employed an ad-hoc questionnaire (H_1-4_). Some aspects of Perceived Catchiness [59] were already measured (positive affect/Pleasure) or difficult to assess with single self-report items (engagement, immediacy). Hence, we operationalized Perceived Catchiness with four items that each correspond to a remaining aspect of catchiness: straightforward catchiness (“This music is catchy.”), perceived memorability [54] (“This music is memorable.”), listener’s interest (“This music sparked my interest.”), and distinctiveness (“This music is distinctive compared to other music that I have heard in the experiment so far.”). Statistical tests (see below) showed that this operationalization was reliable and can be interpreted as measuring a single construct.

For catchiness as memorability, we used a recognition task that tests the memorability of unknown music in two phases [54]. In the memorization phase, participants heard a set of music excerpts. In the recognition phase, they heard a combination of previously heard and new excerpts and indicate whether an excerpt appeared in the previous phase. We converted these yes/no answers to correct/incorrect, hence, we included positive and negative recognition. Completing both phases in one session (as opposed to separated by several hours) required the task to be challenging, hence, we added extra cognitive load to the memorization phase [63–65]. For this purpose, we developed a visual search task that lasted as long as our stimuli (duration 11s to 29s): participants marked iterations of a two-symbol string in a matrix of 20x20 symbols varying in color (red or black) and form (triangle, rhombus, square). See S1 File for an example and details on our piloting.

For the promoting factors hypothesis H_4_, we required several measures. As objective musical factor (H_4a_), we considered the stimulus’ Instrument (Drums, Bass or Keys/Guitar). We measured Perceived Complexity (H_4b_) with a single 7-point Likert item. Previous research has found a quadratic relationship between complexity and PLUMM, therefore, we included a quadratic term for Perceived Complexity. For listener related features (H_4c_), we assessed a participant’s Familiarity with the music (aside from appearances in the experiment) with a forced choice item. We measured participants’ Expertise (four measures condensed into a one-dimensional variable through Confirmatory Factor Analysis) and Dance Preferences (mean of the two items, see above). For Popular Music Affinity, we took the mean of a participant’s liking preferences for 13 popular music styles. Lastly, we measured participants’ taste as Style Bias [33]: participants assigned each stimulus to one of 13 styles for which they provided preferences earlier, and the Style Bias indicates the participant’s liking for the assigned style.

Participants rated their emotional experience in relation to the music with two items (‘Being Moved’). We excluded this measure in the analysis as relating our results to an additional psychological concept is beyond the scope of this paper. S2 File contains the complete questionnaire battery with all items and answer options.

### Stimuli

To assess PLUMM and catchiness, we required music with pronounced rhythmic and melodic aspects. We aimed for unknown music since variation in familiarity is hard to control and potentially confounding in recognition tasks. To fulfill these criteria, we composed 80 8-bar sequences of popular music that each featured Drums, Bass, and Keys or Guitar (grouped as Keys/Guitar). These ecologically valid ‘full’ rhythm section excerpts, consisting of typical patterns of the respective instruments, were created to also suit a follow-up experiment that will examine pattern interactions. However, in the present experiment, we used the 240 individual instrument parts as stimuli (80x Drums, 80x Bass, 80x Keys/Guitar) to focus on PLUMM and catchiness in single instrument patterns as a first step.

The stimuli were designed to represent a random sample of popular music, they are not systematically varied but vary in many aspects. None are intended copies of existing music, but some were inspired by specific tracks. Most patterns are repetitive, some evolve (e.g., through chord progressions), and others include one-time events (e.g., a drum fill). The stimuli were recorded with a midi keyboard on a 2017 MacBook Pro in GarageBand (version 10.4.6). We quantized the rhythm but kept note velocity as performed. We created the mix on rhythm section level (i.e., in relation to the other simultaneous patterns), hence, stimuli loudness is not normalized. We added Reverb to all tracks with GarageBand’s Wooden Verb preset. For each instrument, we created 5 different MIDI sample presets and assigned each pattern to the subjectively most fitting samples. The samples were chosen to represent a broad set of styles, not a specific gerne. More details on the samples can be found in S3 File. The audio stimuli and further information are available at https://osf.io/96qpf/.

### Design

We chose a balanced incomplete design in which each of the 240 stimuli was rated by 30 participants. For a 30-minute experiment, we had 16 stimuli in each phase of the recognition task, and hence needed 450 participants. To keep the design balanced despite drop-outs or exclusions, we pseudo-randomized the order by presenting the same stimuli combination to other participants. We excluded data with identical answers in the recognition task or questionnaires. We screened the final 450 datasets for suspicious data but found none. In an incomplete design recognition task, similar stimuli can be problematic: some participants are challenged by a selection of overly similar stimuli while others are not. Hence, we intended to avoid similar stimuli appearing together. Based on musical notation, we created a list with pairs of potentially similar stimuli using IDyOM (version 1.6) [66]. We sorted this list by ear, as many of the identified pairs are audibly dissimilar (different tempi or samples). As a result, we excluded 44 stimuli pairings.

### Procedure

We set up the experiment on the SoSci Survey platform (www.soscisurvey.de) and recruited participants via Amazon Mechanical Turk (www.mturk.com) between February 4th, 2022 and March 25th, 2022. Participants were informed about the general purpose of the experiment but not about the recognition task. Participants gave written informed consent by clicking on an adequately labeled button. The study was performed in line with the principles of the Declaration of Helsinki. Approval was granted by the University of Birmingham’s Humanities and Social Sciences Ethical Review Committee. Participants were encouraged to use headphones. The experiment consisted of four phases. (1) Participants performed the search task while listening to the stimuli (S1 File). This served to present the stimuli while adding cognitive load and preventing focus on the music directly. Participants familiarized themselves with the task and adjusted the loudness to a comfortable level in an example, then, the same procedure was repeated for 16 stimuli. (2) They answered questions about themselves, including age, gender, country of residence, musical expertise, and music and dancing preferences. (3) Participants then heard 16 stimuli, of which 8 were repeated from (1), each on a separate page. They indicated whether they thought that a stimulus appeared in (1), and whether the music was familiar aside from that. Here, they also rated their experience with questionnaires either on a) Pleasure and Urge to Move or b) Perceived Catchiness, Perceived Complexity and Being Moved. (4) The final phase repeated the stimuli of (3) but presented the respective other questionnaires. We split the PLUMM and Perceived Catchiness questionnaires to different phases to reduce potential positive correlations due to item proximity [67]. Finally, participants were asked to assign the stimuli to one or more of 13 popular music styles (optional). The mean total duration of an experiment was 30 minutes (SD = 10), participants were paid $4.

### Statistical analysis

The statistical analysis was conducted with R (version 4.2.2) in the RStudio environment (version 2022.07.2)

#### Data preparation and factor analysis

We z-scaled and centered all numerical variables to make estimates within regression models comparable [68]. The distribution of questionnaire answers was positively skewed and heavy tailed. The Style Bias variable had 858 missing values because some participants skipped the style assignment. We assumed this data to be missing at random [69] and thus performed multiple imputation based on the Participant ID and the variables Familiarity, Popular Music Affinity, and Expertise with the MICE package [70]. We performed a Confirmatory Factor Analysis with the lavaan package [71] to reduce the dimensions of the ratings, which showed an excellent fit (CFI = 1.000, RMSEA 0.024, 90% CI [0.020, 0.028], SRMR = 0.013). The items had high loadings on the respective factors (0.676–0.911), and Cronbach’s alpha for the ad-hoc Perceived Catchiness questionnaire showed good reliability (α = 0.87, calculated with the psych package [72]). Hence, we proceeded with the factor predicts as condensed ratings for Perceived Complexity, Pleasure, and Urge to Move. For testing hypotheses with a Bayesian approach [73] we used the BayesFactor package [74].

#### Regression models

We chose Bayesian regression models (BRMs) for our analysis (H_1-4_), which we computed with the BRMs package [75–77]. This method combines multivariate regression analysis with Bayesian inference [75, 77]. As setting priors for multivariate models is difficult and our 7200 data were sufficient for a data-driven approach, we set weakly informative priors [78–82]. BRMs had two advantages in our case: we could avoid problems with convergences and statistical power even without excessive amounts of data, and we could perform robust modeling with t-student distributions to cope with the heavy tailed data distribution [83].

In our repeated measures design, the rating data points depend on the stimulus and the participant, which we both regarded as random samples, and hence expected ‘prototypical’ [84] by-participant and by-stimulus random effects. We tested for the optimal model structure empirically by comparing the ELPD (theoretical expected log pointwise predictive density) of models with different terms, including the maximal structure [85], obtained through leave-one-out cross-validation [86, 87] that we computed with the loo package [88]. We performed Pareto smoothed importance sampling to adjust for outlier posterior distributions. With one exception (see below) all selected models featured by-participant and by-stimulus random slopes (see S4 File for more details), indicating that variables affected individual participants differently and showed different effects in individual stimuli. Adjusted *R*^*2*^ were computed using a leave-one-out-adjusted posterior distribution from the performance package [89]. In case of occuring problems, we refitted the respective models with more iterations, more chains, or more robust sampling for converged models with sufficient effective samples sizes for reliable posteriors [75].

#### Causal mediation analysis

Regression models reveal directed effects but are not expedient to investigate a triangle relationship. Hence, we tested the hypothesis of Pleasure as a link between Urge to Move and Perceived Catchiness (H_3_) through mediation analysis. Mediation analysis allows to analyze the causality between three variables: an independent variable, a dependent variable, and a mediator [90, 91]. Three models were calculated to reveal direct (*c’*) and mediated (*ab*) effects between the variables: a model each for the total effect *c* of Catchiness on Urge to Move, the effect *a* of Catchiness on the mediator Pleasure, and for the effects of Catchiness (*c’*) and Pleasure (*b*) on Urge to Move when both were considered. We calculated the required three models with the same random factor structure as our other models. As the random slopes differed between the three models (i.e., *ab* = *c—c’* is not true), we calculated the mediated effect ab = a * b.

## Results

### Recognition and Perceived Catchiness

**Fig 1:**
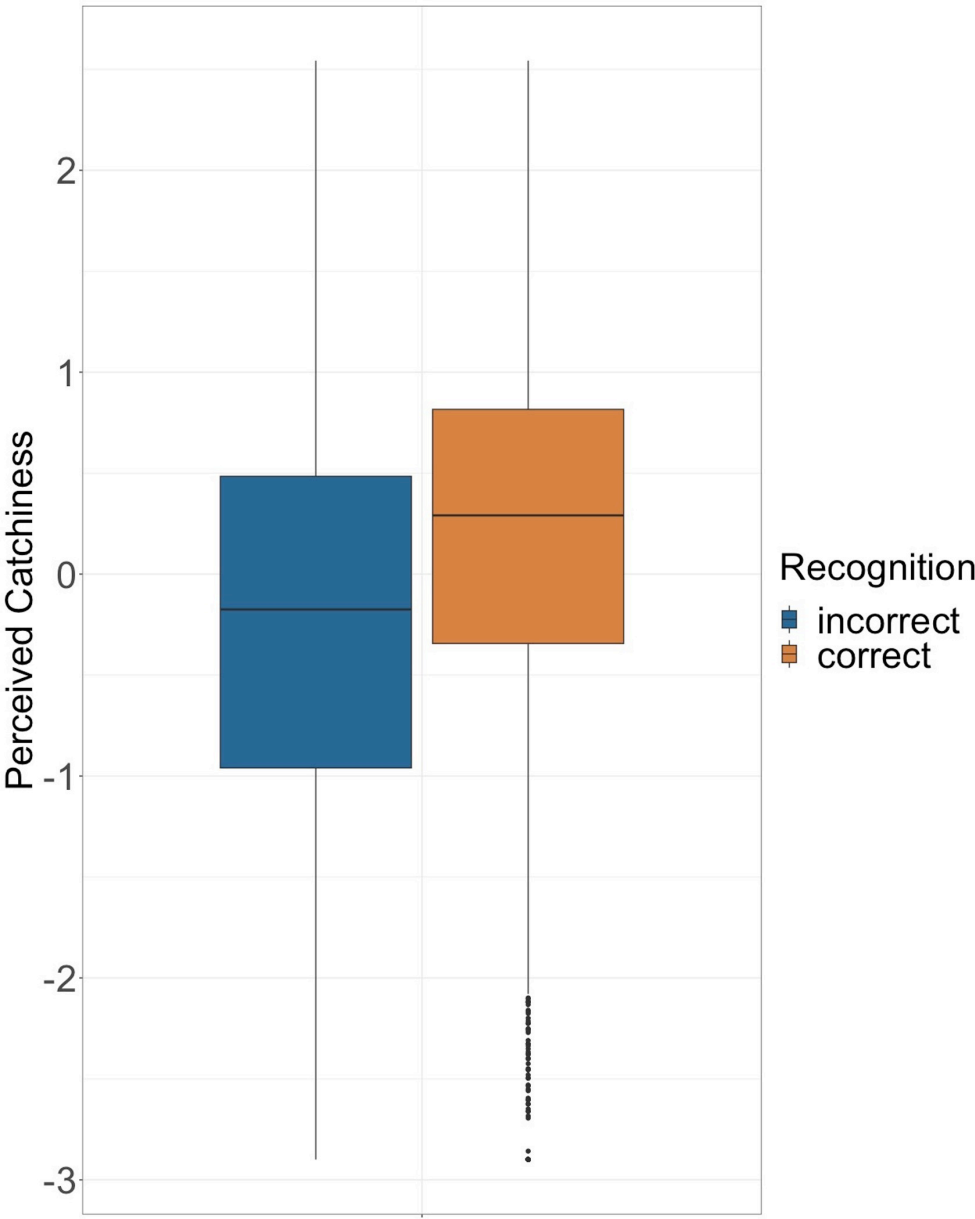
Recognition and Perceived Catchiness. Boxplot with the Perceived Catchiness ratings for correct and incorrect answers in the Recognition task.

Overall, 56% of the answers in the recognition task were correct. This suggests the task was challenging, confirming that the experimental paradigm worked as intended. A Bayesian t-test provided extreme evidence that correct answers in the recognition task were rated higher on Perceived Catchiness than incorrect answers. (BF > 1000, see also Fig 1). Yet, the different instruments suggest a more complex picture: while Drums (60.7%) were recognized more often than Bass (54.7%, BF_Drums–Bass_ = 163) and Keys/Guitar (53.8%, BF_Drums—Keys/Guitar_ > 1000), which in turn performed similarly (BF _Keys/Guitar—Bass_ = 0.028), Keys/Guitar were nominally rated highest on Perceived Catchiness (0.046), but statistically only higher than Bass (-0.065, BF _Keys/Guitar–Bass_ = 41.7), not Drums (0.019, BF_Drums—Keys/Guitar_ = 0.035).

To investigate the relationship further, we computed a univariate regression that predicts Perceived Catchiness from Recognition. This model confirmed that Recognition is positively related with Perceived Catchiness (*b* = 0.262, *SE* = 0.029, 95% CrI [0.205, 0.319]). However, the explained variance is small (*R*^2^_m_ = 0.037, 95% CI [0.031, 0.042]).

### A comprehensive model for Perceived Catchiness

For the comprehensive model for Perceived Catchiness, a random by-stimulus term was unnecessary, despite that an anova showed extreme evidence for an effect of the stimulus on Perceived Catchiness (BF > 1000) on its own. Apparently, the stimulus-dependent predictors in the comprehensive model (Pleasure, Instrument, Style Bias, and Familiarity) provided sufficient explanation in that regard. Overall, the model explained Perceived Catchiness well (*R*^2^_c_ = 0.743 (95% CI [0.728, 0.757]), *R*^2^_m_ = 0.608 (95% CI [0.593, 0.623]). Table 1 lists the model’s fixed effects. Pleasure had a substantial positive effect, while recognition had a considerably smaller effect compared to the univariate model above, as other variables took over some of its explanatory potential.

**Table 1:** Fixed effects of the multivariate Perceived Catchiness model with nine predictors.

	*b*	SE	95% CrI
*Intercept*	-0.067	0.022	-0.111, -0.023
Pleasure	0.437	0.015	0.407, 0467
Recognition	0.068	0.014	0.040, 0.097
Perceived Complexity	0.253	0.016	0.222, 0.284
Perceived Complexity^2	-0.044	0.012	-0.066, -0.021
Instrument: Drums	-0.056	0.017	-0.088, -0.023
Instrument: Keys/Guitar	0.068	0.014	0.041, 0.095
Familiarity	0.116	0.017	0.084, 0.148
Style Bias	0.024	0.007	0.009, 0.038
Popular Music Affinity	0.082	0.021	0.042, 0.126
Dance Preference	0.066	0.019	0.029, 0.104
Expertise	0.035	0.018	0.00, 0.071

Perceived Complexity contributed strongly. The negative quadratic term did not lead to an inverted-U relationship, but a diminishing slope: increasing Perceived Complexity had a strong positive effect on Perceived Catchiness, but for high Perceived Complexity the effect leveled off. All listener-related variables had a positive impact on Perceived Catchiness: Familiarity was most important, followed by Popular Music Affinity and Dance Preferences, with Style Bias and Expertise showing small effects. We found a small overall Instrument bias for Perceived Catchiness: Keys/Guitar were rated as catchier than Bass, and the latter as catchier than Drums.

### Predicting PLUMM with Perceived Catchiness

**Fig 2:**
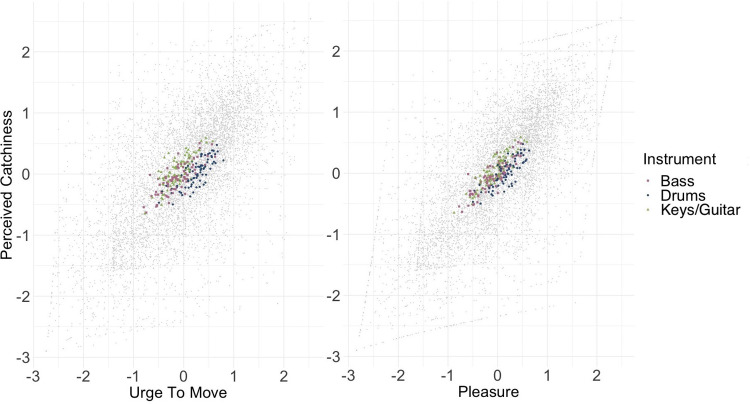
Ratings for Perceived Catchiness, Urge to Move, and Pleasure. Perceived Catchiness is on the y-axis, Urge to Move and Pleasure on the respective x-axes. Grey dots are individual ratings, while coloured symbols represent mean ratings per stimulus: pink squares for Bass, blue dots for Drums, and green triangles for Keys/Guitar.

Fig 2 shows the relationships of Perceived Catchiness with Urge to Move and Pleasure. There is a visible trend towards a linear relationship between Perceived Catchiness and both Urge to Move and Pleasure, as most data points fall into a diagonal oval. The means appear clustered around the middle, but anovas revealed extreme evidence for effects of the individual stimulus on Urge to Move and Pleasure alike (both BFs > 1000).

Our multivariate regression models predicted Urge to Move (R^2^_c_ = 0.650 (95% CI [0.631, 0.668]), R^2^_m_ = 0.502 (95% CI [0.485, 0.519]) and Pleasure (R^2^_c_ = 0.657 (95% CI [0.639,0.675]), R^2^_m_ = 0.566 (95% CI [0.548, 0.583]) similarly well, with stronger fixed effects for Pleasure. Table 2 shows all the fixed effects. The standardized estimates allow for comparisons within each model [68] and, due to similar structure, rough comparisons between the models. Perceived Catchiness had the strongest effect, especially for Pleasure. Correct Recognition of the music influenced both ratings positively. Drum stimuli were associated with higher ratings compared to Bass and Keys/Guitar, especially for Urge to Move. The listener-related variables were all positive, and somewhat stronger for Urge to Move, with Familiarity being most important, followed by Dance Preferences.

**Table 2:** Fixed effects of the multivariate models that predict Urge to Move and Pleasure from the same predictor variables.

	Urge to Move			Pleasure		
	*b*	SE	95% CrI	*b*	SE	95% CrI
*Intercept*	-0.206	0.028	-0.261, -0.150	-0.170	0.025	-0.220, -0.122
Perceived Catchiness	0.401	0.017	0.368, 0.433	0.546	0.016	0.514, 0.578
Recognition	0.072	0.018	0.037, 0.106	0.077	0.018	0.041, 0.113
Instrument: Drums	0.270	0.029	0.215, 0.328	0.158	0.022	0.115, 0.202
Instrument: Keys/Guitar	-0.044	0.024	-0.093, 0.004	-0.014	0.021	-0.056, 0.029
Familiarity	0.164	0.022	0.121, 0.207	0.158	0.021	0.117, 0.200
Dance Preference	0.150	0.023	0.104, 0.196	0.104	0.021	0.062, 0.146
Popular Music Affinity	0.126	0.025	0.078, 0.175	0.097	0.022	0.054, 0.140
Expertise	0.071	0.023	0.027, 0.116	0.039	0.018	0.062, 0.075
Style Bias	0.043	0.010	0.023, 0.063	0.057	0.010	0.037, 0.077

In these models, Perceived Complexity proved redundant (Urge to Move: *b* = 0.015, 95% CrI [-0.008, 0.038]; Pleasure: *b* = -0.003, 95% CrI [-0.026, 0.021]) and was consequently dropped. On its own, it served as a good predictor that showed a positive linear (i.e., there was no evidence for including the quadratic term) relationship with both factors (Urge to Move: *b* = 0.162, *SE* = 0.018, 95% CrI [0.126, 0.198]; Pleasure: *b* = 0.195, *SE* = 0.019, 95% CrI [0.158, 0.232]).

### Mediation analysis

**Fig 3:**
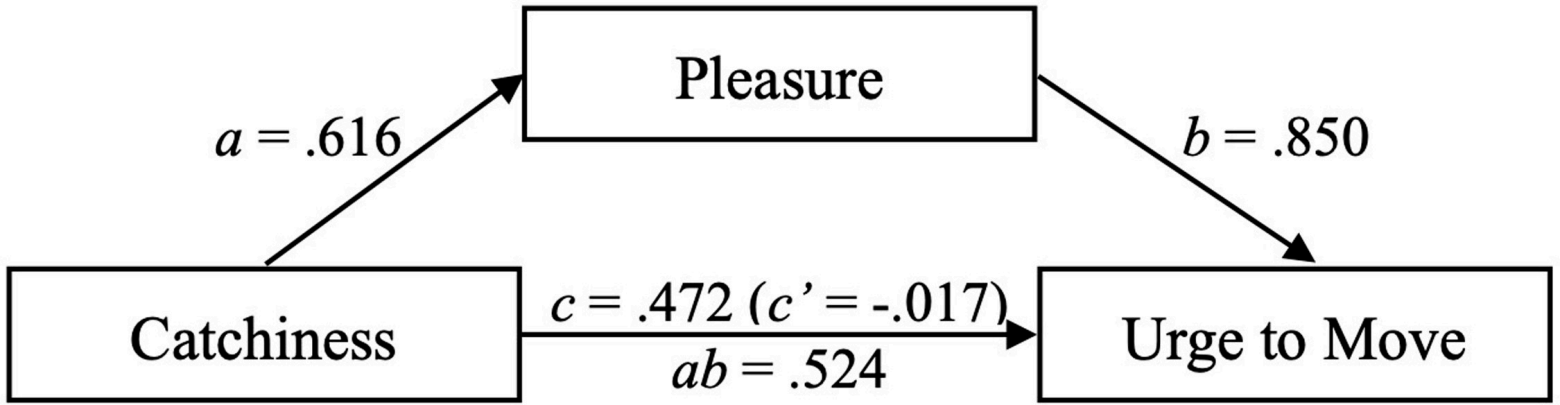
Mediation analysis results. *c* is the total effect, *c’* the direct effect, *ab* the mediated effect.

We found a positive total effect *c* of Perceived Catchiness on Urge to Move (*b* = 0.472, 95% CrI [0.439, 0.506]), a positive effect *a* of Perceived Catchiness on Pleasure (*b* = 0.616, 95% CrI [0.585, 0.647]), a positive effect *b* of Pleasure on Urge to Move (*b* = 0.850, 95% CrI [0.826, 0.874]), and a tiny negative direct effect *c’* of Perceived Catchiness on Urge to Move (*b* = -0.017, 95% CrI [-0.034, -0.000]). We calculated the causal mediation effect *ab* as 0.616 * 0.850 = 0.524 (Fig 3).

The opposed direction of *c’* compared to *ab* and *c* indicated negative confounding: Pleasure suppressed Perceived Catchiness’ effect on Urge to Move [92]. However, the CrI of *c’* suggested potential redundancy. Hence, we compared our model that considered Pleasure and Perceived Catchiness against a null model with Pleasure as sole predictor. As above, we calculated the theoretical expected log pointwise predictive density (ELPD) based on leave-one-out cross validation of the two models. The null model’s ELPD was higher by 86.8, confirming that *c’* was not negligible and a valuable addition.

## Discussion

Our results mostly supported our four hypotheses. We found a positive relationship between Perceived Catchiness and PLUMM (H_2_). More specifically, we found that the effect of Perceived Catchiness on the Urge to Move to music is moderated by Pleasure, supporting the pleasure as hinge hypothesis H_3_. We added to the understanding of catchiness by providing evidence for a distinction between Perceived Catchiness and Recognition, a strong effect of Pleasure and thus multi-dimensionality (H_1_). We identified factors that lead to Perceived Catchiness and PLUMM alike, and others with diverging effects (H_4_). As findings about catchiness are a precondition for other implications of this study, we first discuss catchiness before turning to how it relates to PLUMM and groove.

### On catchiness

We found a positive relationship between Recognition and Perceived Catchiness, but it was weak (like recognition and perceived memorability in Russell [54]). Hence, our results do not support regarding them as the same phenomenon nor closely related. We found a strong effect of Pleasure on Perceived Catchiness, supporting Bechtold et al.’s theory [59] that positive affect is a central to catchiness (which may partly explain the “vital role” [40] (p.183) of catchiness in popular music). Taken together, this means that the Perceived Catchiness that we find in daily parlance (“this music is catchy”) has more dimensions besides the memorability of music, supporting a similar conclusion by Bechtold et al. [59] and our H_1_. Our operationalization of Perceived Catchiness intertwined catchiness with perceived memorability, listeners’ interest, and the music’s perceived distinctiveness. As these are all likely to affect recognition and salience as well, our results do not per se contradict Burgoyne et al.’s definition [41] of catchiness as long-term musical salience but expand it. Future research is needed to disentangle and quantify the complex web of interactions and relationships that can be hypothesized around different dimensions of catchiness. Yet, our results suggests that future studies need to be clear whether they measure catchiness as memorability (with findings restricted to that dimension) or whether they investigate catchiness more holistically, as a multi-dimensional perceived musical quality.

For Perceived Complexity, our results suggest that catchy music requires a certain complexity, contradicting Russell’s findings for catchiness as memorability [54]. This finding has sense to it: simple music is easy to memorize due to low amount of information, but may lack qualities that make it interesting, distinctive, and catchy. On the other end of the spectrum, we could not support the theory of Bechtold et al. [59] that music can be (perceived as) too complex to be catchy, potentially because our stimuli (consisting of realistic and short single instrument patterns) were not complex enough for this to occur within our experiment.

Our results emphasize the subjectivity of catchiness. We found positive effects of listener-related variables, in particular Familiarity, Popular Music Affinity and Dance Preferences, but also Style Bias and Expertise. These results sit well with the importance of individual differences for involuntary musical imagery [49, 93, 94]. In comparison, the Instrument, as objective musical quality, had a small effect. Hence, this partially supports Burgoyne et al.’s assumption that some music is inherently catchier than other music [41] but adds that listeners’ backgrounds are vital for understanding catchiness. A future study that focuses on the interaction of musical-, listener-, and situation-related factors could shed more light on why some music is perceived as catchier than others.

### The relationships of Perceived Catchiness, PLUMM, and groove

Our results show a strong effect of Perceived Catchiness on Urge to Move and Pleasure ratings alike, even when listener- and music-related variables are considered. Hence, we can say that music perceived as catchy is likely to induce PLUMM, confirming the positive relation hypothesis H_2_. But the relationship is complex. While Perceived Catchiness is associated with an increased Urge to Move, our analysis shows an indirect effect that is almost fully suppressed by Pleasure: the latter explains all the variance that Perceived Catchiness explains, except for a tiny bit, which perhaps could be more substantial without a potential proximity bias between Pleasure and Urge to Move. Yet, even in this case, catchiness acts as a corrective that adds to the predictive validity of Pleasure on Urge to Move. These findings support our theory that Pleasure is a causal link between catchiness and the urge to move (H_3_).

What does the relationship between catchiness and PLUMM mean for groove and for future studies? As PLUMM is central to groove, it is likely that Perceived Catchiness is also a good predictor for groove. Whether Perceived Catchiness could be a dimension of groove remains unclear. Potentially, it could be an associated mental process and as such incorporated into the Psychological Groove Model [37–39], or included as predictor in other models. However, because of the exploratory nature of our study, we can only speculate about this, as we require more research into the mechanics of how catchiness influences groove, for example with experiments that control for either Perceived Catchiness, Pleasure, or the Urge to Move to investigate independence in this triumvirate.

### On PLUMM

Our results corroborate earlier findings for the PLUMM related to drum patterns [33, 34]. The redundancy of Perceived Complexity is a surprising result, as (objective) complexity proved important in previous studies [17–24]. Presumably, Perceived Catchiness took over Perceived Complexity’s effect in our models (exacerbated by a potential proximity bias between the two). Yet, catchiness cannot replace complexity as a factor for groove, as they are clearly different concepts and, at least in our data, related non-linearly. As above, we can speculate that we did not find an inverted-U between Perceived Complexity and PLUMM because our stimuli, composed to portray common popular music, did not cover the full range of complexity to captures this effect.

### Comparing factors for Perceived Catchiness and PLUMM

As we considered the same predictors for Perceived Catchiness, Urge to Move, and Pleasure, we can examine which overlap and which differ (H_4_). However, as the results are based on separate albeit similarly structured models, any comparison is restricted to a superficial level (e.g., we cannot compare strengths of effects).

In line with H_4a_, the musical properties lead to reverse results: drum patterns show a positive bias for PLUMM, but a negative one for Perceived Catchiness, and vice versa for Keys/Guitar. Hence, the musical content is potentially complicating the relationship between PLUMM and Perceived Catchiness. In contrast to our expectation of opposed effects (H_4b_), Perceived Complexity showed positive influences for both, but it was linear for PLUMM and quadratic for Perceived Catchiness. The recognizability of the music influenced all three ratings positively. We expected this for Perceived Catchiness and note that even in its understanding purely as memorability, catchiness positively affects PLUMM. The listener-related variables in all models unanimously show positive effects, i.e., they all promote PLUMM and Perceived Catchiness, confirming H_4c_. This overlap can partly explain why catchiness and groove appear together frequently. We can hypothesize a causality in some cases: catchy music is easier to familiarize with, and Familiarity leads to higher PLUMM. In summary, we can conclude for our promoting factor hypothesis (H_4_) that the listener-related (Familiarity, preferences and Expertise), behavioral (Recognition), and subjective (Perceived Complexity) factors overlap for PLUMM and Perceived Catchiness but the musical factors differ.

### Limitations

Limitations for this study include potential issues with proximity biases between Perceived Catchiness-Perceived Complexity, and Pleasure-Urge to Move. Further, our results are limited to short and rather simple stimuli, and cannot directly be applied to more multi-layered, longer, or more complex music. We have ignored other potentially common factors, such as the spatial and mental listening situation. Accounting for this by, e.g., measuring participants’ current mood or mental state could strengthen the models and might reveal another overlap between PLUMM and Perceived Catchiness. And lastly, we restricted the groove experience to the PLUMM aspect, which means that we were able to show a positive relationship between catchiness and that specifically, but can only speculate about groove experiences as a whole.

## Conclusion

In this study, we pursued several questions about the relationship between the experience of groove and catchiness. In a first step, we substantiated the notion that catchiness encompasses more than memorability and is also subjective. We found a general trend that catchy music is more likely to afford an urge to move than less catchy music. Our analysis showed that pleasure is important for perceived catchiness and the urge to move alike, and understanding pleasure as a hinge explains the positive effect between them. Comparisons between causes for PLUMM and perceived catchiness suggested that the same listener-related factors influence our experiences of these phenomena. In contrast, the actual musical content had diverging effects, indicating that musical causes for PLUMM and catchiness differ.

The result that groove and catchiness are related and influence each other positively is a major and novel finding. Our results allow to connect these two very common psychological phenomena that play a central role in popular music and provide a first quantitative glimpse of how these interact. As a next step, we will examine the relationship between groove and catchiness in a more complex musical environment with several instruments playing simultaneously, combining patterns of different PLUMM and catchiness levels (as our stimuli are already geared towards that), and thus investigate their relationship in more systematic ways.

## Supporting information

S1 File Piloting and search task. (DOCX)

S2 File Questionnaire battery. (DOCX)

S3 File Stimuli samples. Details on the samples used for the stimuli creation.
(DOCX)

S4 File Model selection. Results of the model selection processes.
(DOCX)

## References

[1] Janata P, Tomic ST, Haberman JM. Sensorimotor coupling in music and the psychology of the groove. J Exp Psychol Gen. 2012;141(1):54–75. doi: 10.1037/a0024208 21767048

[2] Pando-Naude V, Matthews TE, Højlund A, Jakobsen S, Østergaard K, Johnsen E, et al. Dopamine dysregulation in Parkinson’s disease flattens the pleasurable urge to move to musical rhythms. bioRxiv [preprint]. 2023 [cited 2023 Oct 31]. p. 2023.02.27.530174. Available from: https://www.biorxiv.org/content/10.1101/2023.02.27.530174v2 doi: 10.1111/ejn.16128 37724707

[3] Matthews TE, Stupacher J, Vuust P. The pleasurable urge to move to music through the lens of learning progress. J Cogn. 2023;6(1):55. doi: 10.5334/joc.320 37720891 PMC10503533

[4] Hosken F. The subjective, human experience of groove: A phenomenological investigation. Psychology of Music. 2020;48(2):182–98. 10.1177/0305735618792440

[5] Stupacher J, Bechtold T, Senn O. A text mining approach to the use of “groove” in everyday language. Psychology of Music. 2023 Oct 27;03057356231205883. 10.1177/03057356231205883

[6] Pfleiderer M. Dimensionen der Groove-Erfahrung: Eine empirische Studie. Popscriptum. 2010;(11). 10.18452/20300. German.

[7] Danielsen A. Presence and pleasure: The funk grooves of James Brown and Parliament. Middletown: Wesleyan University Press; 2006.

[8] Duman D, Snape N, Danso A, Toiviainen P, Luck G. Groove as a multidimensional participatory experience. Psychology of Music. 2023;1–24. 10.1177/03057356231165327

[9] Câmara GS, Danielsen A. Groove. The Oxford handbook of critical concepts in music theory. 2018. 10.1093/oxfordhb/9780190454746.013.17

[10] Etani T, Miura A, Kawase S, Fujii S, Keller PE, Vuust P, et al. A review of psychological and neuroscientific research on musical groove. Neuroscience & Behavioral Reviews. 2024. doi: 10.1016/j.neubiorev.2023.105522 38141692

[11] Frühauf J, Kopiez R, Platz F. Music on the timing grid: the influence of microtiming on the perceived groove quality of a simple drum pattern performance. Musicae Scientiae. 2013;17(2):246–60. 10.1177/1029864913486793

[12] Davies M, Madison G, Silva P, Gouyon F. The effect of microtiming deviations on the perception of groove in short rhythms. Music Perception: An Interdisciplinary Journal. 2013;30(5):497–510. 10.1525/mp.2013.30.5.497

[13] Senn O, Kilchenmann L, von Georgi R, Bullerjahn C. The effect of expert performance microtiming on listeners’ experience of groove in swing or funk music. Front Psychol. 2016;7(1487):1–16. doi: 10.3389/fpsyg.2016.01487 27761117 PMC5050221

[14] Datseris G, Ziereis A, Albrecht T, Hagmayer Y, Priesemann V, Geisel T. Microtiming deviations and swing feel in jazz. Sci Rep. 2019;9(1):19824. doi: 10.1038/s41598-019-55981-3 31882842 PMC6934603

[15] Câmara GS, Nymoen K, Lartillot O, Danielsen A. Effects of instructed timing on electric guitar and bass sound in groove performance. The Journal of the Acoustical Society of America. 2020;147(2):1028–41. doi: 10.1121/10.0000724 32113267

[16] Câmara GS, Nymoen K, Lartillot O, Danielsen A. Timing is everything…or is it? Effects of instructed timing style, reference, and pattern on drum kit sound in groove-based performance. Music Perception. 2020;38(1):1–26. 10.1525/mp.2020.38.1.1

[17] Park S. Effects of microtiming deviations between two instruments on the groove experience. [Internet]. 2021 [cited 2023 Sep 21]; Available from: https://koasas.kaist.ac.kr/handle/10203/295116

[18] Witek MAG, Clarke EF, Wallentin M, Kringelbach ML, Vuust P. Syncopation, body-movement and pleasure in groove music. PLOS ONE. 2014;9(4): e94446 doi: 10.1371/journal.pone.0094446 24740381 PMC3989225

[19] Madison G, Sioros G. What musicians do to induce the sensation of groove in simple and complex melodies, and how listeners perceive it. Front Psychol. 2014;5. 10.3389/fpsyg.2014.00894 25191286 PMC4137755

[20] Sioros G, Miron M, Davies M, Gouyon F, Madison G. Syncopation creates the sensation of groove in synthesized music examples. Front Psychol. 2014;5:1–10. 10.3389/fpsyg.2014.01036 25278923 PMC4165312

[21] Witek MAG, Popescu T, Clarke EF, Hansen M, Konvalinka I, Kringelbach ML, et al. Syncopation affects free body-movement in musical groove. Exp Brain Res. 2017;235(4):995–1005. doi: 10.1007/s00221-016-4855-6 28028583

[22] Matthews TE, Witek MAG, Heggli OA, Penhune VB, Vuust P. The sensation of groove is affected by the interaction of rhythmic and harmonic complexity. PLOS ONE. 2019;14(1):e0204539. doi: 10.1371/journal.pone.0204539 30629596 PMC6328141

[23] Stupacher J, Matthews TE, Pando-Naude V, Foster Vander Elst O, Vuust P. The sweet spot between predictability and surprise: musical groove in brain, body, and social interactions. Frontiers in Psychology. 2022;13(906190):1–9. doi: 10.3389/fpsyg.2022.906190 36017431 PMC9396343

[24] Stupacher J, Wrede M, Vuust P. A brief and efficient stimulus set to create the inverted U-shaped relationship between rhythmic complexity and the sensation of groove. PLOS ONE. 2022;17(5):e0266902. doi: 10.1371/journal.pone.0266902 35588097 PMC9119456

[25] Spiech C, Sioros G, Endestad T, Danielsen A, Laeng B. Pupil drift rate indexes groove ratings. Sci Rep. 2022;12(1):11620. doi: 10.1038/s41598-022-15763-w 35804069 PMC9270355

[26] Céspedes-Guevara J, Witek MAG. Syncopation levels, but not movement, are associated with pleasantness while listening to rhythmic music. Psychology of Music. 2023;03057356231153062. 10.1177/03057356231153062

[27] Wesolowski BC, Hofmann A. There’s more to groove than bass in electronic dance music: why some people won’t dance to techno. PLOS ONE. 2016;11(10):e0163938. doi: 10.1371/journal.pone.0163938 27798645 PMC5087899

[28] Hove MJ, Martinez SA, Stupacher J. Feel the bass: music presented to tactile and auditory modalities increases aesthetic appreciation and body movement. Journal of Experimental Psychology: General. 2020;149(6):1137–47. doi: 10.1037/xge0000708 31697113

[29] Lustig E, Tan I. All about that bass: audio filters on basslines determine groove and liking in electronic dance music. Psychology of Music. 2019;0305735619836275. 10.1177/0305735619836275

[30] Cameron DJ, Dotov D, Flaten E, Bosnyak D, Hove MJ, Trainor LJ. Undetectable very-low frequency sound increases dancing at a live concert. Current Biology. 2022;32(21):R1222–3. doi: 10.1016/j.cub.2022.09.035 36347227

[31] Dahl S, Huron D, Brod G, Altenmüller E. Preferred dance tempo: does sex or body morphology influence how we groove? Journal of New Music Research. 2014;43(2):214–23. 10.1080/09298215.2014.884144

[32] Etani T, Marui A, Kawase S, Keller P. Optimal tempo for groove: Its relation to directions of body movement and Japanese nori. Front Psychol. 2018;9. 10.3389/fpsyg.2018.00462 29692747 PMC5902701

[33] Senn O, Kilchenmann L, Bechtold T, Hoesl F. Groove in drum patterns as a function of both rhythmic properties and listeners’ attitudes. PLOS ONE. 2018;13(6):e0199604. doi: 10.1371/journal.pone.0199604 29958289 PMC6025871

[34] Senn O, Bechtold TA, Hoesl F, Kilchenmann L. Taste and familiarity affect the experience of groove in popular music. Musicae Scientiae. 2021;1–22. 10.1177/1029864919839172

[35] Kowalewski DA, Kratzer TM, Friedman RS. Social music: investigating the link between personal liking and perceived groove. Music Perception. 2020;37(4):339–46. 10.1525/mp.2020.37.4.339

[36] Witek MAG, Liu J, Kuubertzie J, Yankyera AP, Adzei S, Vuust P. A critical cross-cultural study of sensorimotor and groove responses to syncopation among Ghanaian and American university students and staff. Music Perception. 2020;37(4):278–97. 10.1525/mp.2020.37.4.278

[37] Senn O, Rose D, Bechtold T, Kilchenmann L, Hoesl F, Jerjen R, et al. Preliminaries to a psychological model of musical groove. Front Psychol. 2019;10(1228):1–5. doi: 10.3389/fpsyg.2019.01228 31214069 PMC6558102

[38] Senn O, Bechtold T, Hoesl F, Jerjen R, Kilchenmann L, Rose D, et al. An SEM approach to validating the psychological model of musical groove. Journal of Experimental Psychology: Human Perception and Performance. 2023;49(3):290–305. doi: 10.1037/xhp0001087 36931839

[39] Senn O, Bechtold TA, Jerjen R, Kilchenmann L, Hoesl F. Three psychometric scales for groove research: inner representation of temporal regularity, time-related interest, and energetic arousal. Music & Science. 2023;6:20592043231185663. 10.1177/20592043231185663

[40] Van Balen JMH. Audio description and corpus analysis of popular music [dissertation]. Utrecht University; 2016.

[41] Burgoyne JA, Bountouridis D, van Balen JMH, Honing H. Hooked: a game for discovering what makes music catchy. In: Proceedings of the 14th Society of Music Information Retrieval Conference (ISMIR). 2013.

[42] Burns G. A Typology of ‘hooks’ in popular records. Popular Music. 1987;6:1–20. 10.1017/S0261143000006577

[43] Honing H. Lure(d) into listening: The potential of cognition-based music information retrieval. Empirical Musicology Review. 2010;5(4):146–51.

[44] Byron T, O’Regan J. Hooks in popular music. Springer Nature; 2022. 10.1007/978-3-031-19000-1

[45] Beaman CP, Williams TI. Earworms (stuck song syndrome): towards a natural history of intrusive thoughts. British Journal of Psychology. 2010;101(4):637–53. doi: 10.1348/000712609X479636 19948084

[46] Byron TP, Fowles LC. Repetition and recency increases involuntary musical imagery of previously unfamiliar songs. Psychology of Music. 2015;43(3):375–89. 10.1177/0305735613511506

[47] Moeck EK, Hyman Jr. IE, Takarangi MKT. Understanding the overlap between positive and negative involuntary cognitions using instrumental earworms. Psychomusicology: Music, Mind, and Brain. 2018;28(3):164–77. 10.1037/pmu0000217

[48] Floridou GA, Williamson VJ, Stewart L, Müllensiefen D. The involuntary musical imagery scale (IMIS). Psychomusicology: Music, Mind, and Brain. 2015;25(1):28–36. 10.1037/pmu0000067

[49] Janetschek S, Frieler K, Lothwesen K. Wahrnehmung und Erleben von Ohrwürmern bei Musikstudierenden und Nicht-Musikstudierenden: Zusammenhänge mit Arbeitsgedächtnis, Tonhöhenvorstellung und musikalischer Erfahrenheit. Jahrbuch Musikpsychologie. 2022;31:1–20. German. 10.5964/jbdgm.165

[50] Campbell S, Margulis EH. Catching an earworm through movement. Journal of New Music Research. 2015;44(4):347–58. 10.1080/09298215.2015.1084331

[51] Accidents Kronengold C., hooks and theory. Popular Music. 2005;24(3). 10.1017/S0261143005000589

[52] Traut D. ‘Simply Irresistible’: recurring accent patterns as hooks in mainstream 1980s music. Popular Music. 2005;24(01):57–77. 10.1017/S0261143004000303

[53] Hook Hume A., line and sinker: how songwriters get into your head. PORESO 2015: Redefining the boundaries of the ‘Event’. 2017;

[54] Russell PA. Memory for music: A study of musical and listener factors. British Journal of Psychology. 1987;78:335–47. 10.1111/j.2044-8295.1987.tb02251.x

[55] Jakubowski K, Finkel S, Stewart L, Müllensiefen D. Dissecting an earworm: melodic features and song popularity predict involuntary musical imagery. Psychology of Aesthetics, Creativity, and the Arts. 2017;11(2):122–35. 10.1037/aca0000090

[56] Scott D, Berry D, Bobbett K. How earworms are born: an EEG study of original melodies that may come to stick in the brain. [Internet]. 2020 [cited 2023 Sep 21]; Available from: https://osf.io/6bhwx/

[57] Korsmit IR, Burgoyne JA, Honing H. If you wanna be my lover … A hook discovery game to uncover individual differences in long-term musical memory. In: Proceedings of the 25th Anniversary Conference of the European Society for the Cognitive Sciences of Music. Ghent; 2017.

[58] Pawley A, Müllensiefen D. The science of singing along: A quantitative field study on sing-along behavior in the north of England. Music Perception. 2012;30(2):129–46. 10.1525/mp.2012.30.2.129

[59] Bechtold TA, Kilchenmann L, Curry B, Witek MAG. Understanding the relationship between catchiness and groove: a qualitative study with popular music creators. Music Perception. 2023;40(5):353–72. 10.1525/mp.2023.40.5.353

[60] Müllensiefen D, Gingras B, Musil J, Stewart L. The musicality of non-musicians: an index for assessing musical sophistication in the general population. PLoS ONE. 2014 Feb 26;9(2):e89642. doi: 10.1371/journal.pone.0089642 24586929 PMC3935919

[61] Senn O, Bechtold T, Rose D, Câmara GS, Düvel N, Jerjen R, et al. Experience of groove questionnaire: instrument development and initial validation. Music Perception. 2020;38(1):46–65. 10.1525/mp.2020.38.1.46

[62] Düvel N, Labonde P, Bechtold T, Senn O, Kopiez R. Experience of groove questionnaire: german translation and validation. Music Perception. 2021;39(1):83–99. 10.1525/mp.2021.39.1.83

[63] Mattys SL, Wiget L. Effects of cognitive load on speech recognition. Journal of Memory and Language. 2011;65(2):145–60. 10.1016/j.jml.2011.04.004

[64] Mitterer H, Mattys SL. How does cognitive load influence speech perception? An encoding hypothesis. Attention, Perception, & Psychophysics. 2017;79(1):344–51. doi: 10.3758/s13414-016-1195-3 27604285

[65] Léveillé Gauvin H. Drawing listener attention in popular music: testing five musical features arising from the theory of attention economy. Musicae Scientiae. 2017;1029864917698010. 10.1177/1029864917698010

[66] Pearce MT. Statistical learning and probabilistic prediction in music cognition: mechanisms of stylistic enculturation. Annals of the New York Academy of Sciences. 2018;Special Issue: The Neurosciences and Music VI:378–95. doi: 10.1111/nyas.13654 29749625 PMC6849749

[67] Podsakoff PM, MacKenzie SB, Lee JY, Podsakoff NP. Common method biases in behavioral research: a critical review of the literature and recommended remedies. Journal of Applied Psychology. 2003;88(5):879–903. doi: 10.1037/0021-9010.88.5.879 14516251

[68] Gelman A, Hill J, Vehtari A. Regression and other stories. Cambridge University Press; 2020.

[69] Bhaskaran K, Smeeth L. What is the difference between missing completely at random and missing at random? International Journal of Epidemiology. 2014 Aug 1;43(4):1336–9. doi: 10.1093/ije/dyu080 24706730 PMC4121561

[70] Buuren S van, Groothuis-Oudshoorn K. mice: multivariate imputation by chained equations in R. Journal of Statistical Software. 2011;45:1–67. 10.18637/jss.v045.i03

[71] Rosseel Y. lavaan: An R package for structural equation modeling. Journal of Statistical Software. 2012 May 24;48:1–36. 10.18637/jss.v048.i02

[72] Revelle W. psych: procedures for psychological, psychometric, and personality research [software]. 2023 [cited 2023 Sep 21]. Available from: https://cran.r-project.org/web/packages/psych/index.html

[73] Morey RD, Rouder JN. Bayes factor approaches for testing interval null hypotheses. Psychological Methods. 2011;16(4):406–19. doi: 10.1037/a0024377 21787084

[74] Morey RD, Rouder JN, Jamil T, Urbanek S, Forner K, Ly A. BayesFactor: computation of Bayes factors for common designs [software]. 2022 [cited 2023 Sep 21]. Available from: https://cran.r-project.org/web/packages/BayesFactor/index.html

[75] Bürkner PC. brms: An R Package for Bayesian multilevel models using stan. Journal of Statistical Software. 2017;80:1–28. 10.18637/jss.v080.i01

[76] Bürkner PC. Advanced Bayesian multilevel modeling with the R Package brms. The R Journal. 2018;10(1):395–411. 10.32614/RJ-2018-017

[77] Bürkner PC, Gabry J, Vehtari A. Efficient leave-one-out cross-validation for Bayesian non-factorized normal and Student-t models. Comput Stat. 2021;36(2):1243–61. 10.1007/s00180-020-01045-4

[78] Lemoine NP. Moving beyond noninformative priors: why and how to choose weakly informative priors in Bayesian analyses. Oikos. 2019;128(7):912–28. 10.1111/oik.05985

[79] McElreath R. Statistical Rethinking: A Bayesian course with examples in R and Stan. Boca Raton: Chapman and Hall/CRC; 2016. 10.1201/9781315372495

[80] Winter B, Bürkner PC. Poisson regression for linguists: A tutorial introduction to modelling count data with brms. Language and Linguistics Compass. 2021;15(11):e12439. 10.1111/lnc3.12439

[81] Gelman A, Simpson D, Betancourt M. The prior can often only be understood in the context of the likelihood. Entropy. 2017;19(10):555. 10.3390/e19100555

[82] Schad DJ, Nicenboim B, Bürkner PC, Betancourt M, Vasishth S. Workflow techniques for the robust use of bayes factors. Psychological Methods. 2022; doi: 10.1037/met0000472 35266787

[83] Juárez MA, Steel MFJ. Model-based clustering of non-Gaussian panel data based on skew-t distributions. Journal of Business & Economic Statistics. 2010;28(1):52–66. 10.1198/jbes.2009.07145

[84] Baayen RH, Davidson DJ, Bates DM. Mixed-effects modeling with crossed random effects for subjects and items. Journal of memory and language. 2008;59(4):390–412. 10.1016/j.jml.2007.12.005

[85] Barr DJ, Levy R, Scheepers C, Tily HJ. Random effects structure for confirmatory hypothesis testing: Keep it maximal. Journal of Memory and Language. 2013;68(3):255–78. doi: 10.1016/j.jml.2012.11.001 24403724 PMC3881361

[86] Vehtari A, Gelman A, Gabry J. Practical Bayesian model evaluation using leave-one-out cross-validation and WAIC. Stat Comput. 2017;27(5):1413–32. 10.1007/s11222-016-9696-4

[87] Sivula T, Magnusson M, Matamoros AA, Vehtari A. Uncertainty in Bayesian leave-one-out cross-validation based model comparison [preprint]. arXiv; 2022 [cited 2024 Mar 26]. Available from: http://arxiv.org/abs/2008.10296

[88] Vehtari A, Gabry J, Magnusson M, Yao Y, Bürkner PC, Paananen T, et al. loo: efficient leave-one-out cross-validation and WAIC for Bayesian models [software]. 2023 [cited 2023 Sep 21]. Available from: https://cran.r-project.org/web/packages/loo/index.html

[89] Lüdecke D, Ben-Shachar MS, Patil I, Waggoner P, Makowski D. performance: an R package for assessment, comparison and testing of statistical models. Journal of Open Source Software. 2021;6(60):3139. 10.21105/joss.03139

[90] Baron RM, Kenny DA. The moderator–mediator variable distinction in social psychological research: Conceptual, strategic, and statistical considerations. Journal of Personality and Social Psychology. 1986;51(6):1173–82. doi: 10.1037//0022-3514.51.6.1173 3806354

[91] MacKinnon D. Introduction to Statistical Mediation Analysis. Routledge; 2012.

[92] MacKinnon DP, Krull JL, Lockwood CM. Equivalence of the mediation, confounding and suppression Effect. Prev Sci. 2000;1(4):173–81. doi: 10.1023/a:1026595011371 11523746 PMC2819361

[93] Liikkanen LA, Jakubowski K. Involuntary musical imagery as a component of ordinary music cognition: A review of empirical evidence. Psychon Bull Rev. 2020;27(6):1195–217. doi: 10.3758/s13423-020-01750-7 32583211 PMC7704448

[94] Hyman IE, Burland NK, Duskin HM, Cook MC, Roy CM, McGrath JC, et al. Going gaga: investigating, creating, and manipulating the song stuck in my head. Applied Cognitive Psychology. 2013;27(2):204–15. 10.1002/acp.2897

