# Supplementary material for: The perceived catchiness of music affects the experience of groove
Source: PLoS One. 2024 May 15;19(5):e0303309. doi: 10.1371/journal.pone.0303309 (PMC11095763; doi:10.1371/journal.pone.0303309)

**S1 File. Piloting and an example search task.**

**Piloting**

For our recognition task, we piloted the time delay between the memorization and recognition phases with the goal of avoiding that participants had to leave and come back for the recognition phase on a different day. A first pilot with six participants and 15 minutes delay between phases showed a ceiling effect as almost all participants correctly recognized the repeated excerpts. Léveillé Gauvin [1] argued that the fight for attention during the encoding process is the bottleneck for memorizing music. Following this and others [2-3], we added extra cognitive load and distraction to the memorization phase. In contrast to previous studies [2-3], we required a relatively long search task for our stimuli (duration 11s to 29s): participants mark iterations of a two-symbol string in a matrix of 20x20 symbols varying in color (red or black) and form (triangle, rhombus, square). We set the number of total symbols in a way that allows participants to find several iterations but never feel certain that they found all.

A second pilot experiment with 23 participants examined the utility of this search task in combination with the recognition task. Ratios for correct recognitions per stimulus were between 0.565 and 0.956 (mean = 0.743, SD = 0.124). Hence, the extra load was well dosed, and made the recognition experiment viable in a single session.

1. Léveillé Gauvin H. Drawing listener attention in popular music: Testing five musical features arising from the theory of attention economy. Musicae Scientiae. 2017;1029864917698010. <https://doi.org/10.1177/1029864917698010>
2. Mattys SL, Wiget L. Effects of cognitive load on speech recognition. Journal of Memory and Language. 2011;65(2):145–60. <https://doi.org/10.1016/j.jml.2011.04.004>
3. Mitterer H, Mattys SL. How does cognitive load influence speech perception? An encoding hypothesis. Attention, Perception, & Psychophysics. 2017;79(1):344–51. <https://doi.org/10.3758/s13414-016-1195-3>

**Example search task**

(Note: the matrix of symbols was hidden until participants clicked on the start button.)

In this task we ask you to find this combination of symbols in the picture below until the music stops playing. Please mark the iterations by clicking anywhere on or between the symbols.


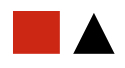


Start


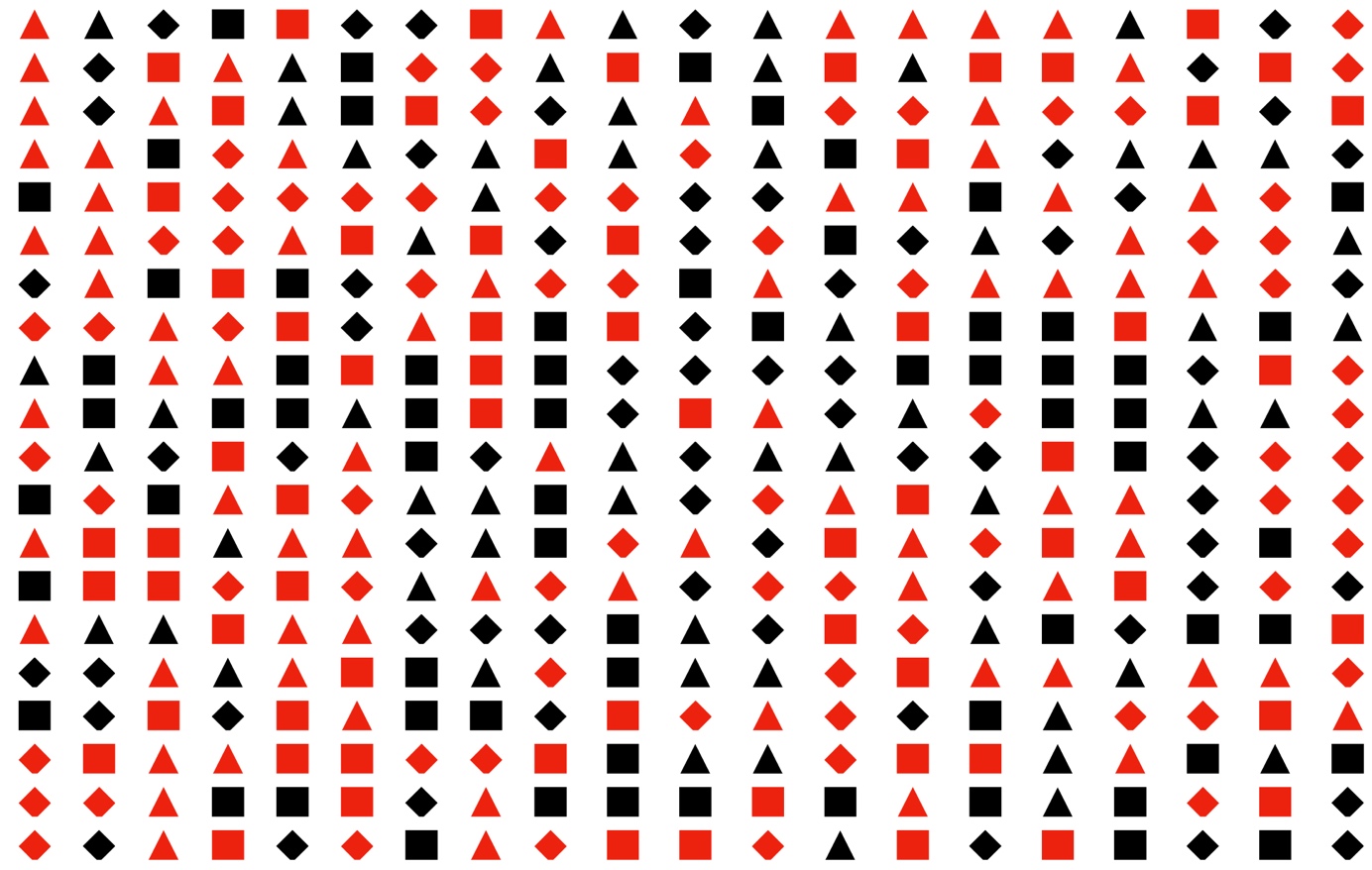

Supplement: S1 File — (DOCX) [file pone.0303309.s001.docx]
