# Supplementary material for: The perceived catchiness of music affects the experience of groove
Source: PLoS One. 2024 May 15;19(5):e0303309. doi: 10.1371/journal.pone.0303309 (PMC11095763; doi:10.1371/journal.pone.0303309)
Supplement: S2 File — (DOCX) [file pone.0303309.s002.docx]

**S2 File. Questionnaire battery.**

1. Recognition

- Have you heard this music during phase I?

yes/no

1. Familiarity

- Aside from a possible appearance during phase I, does this music sound familiar to you?

yes/no

1. Rating scales

For each:

How much do you agree with the following statements?

7-point Likert scales from strongly disagree to strongly agree

Perceived Catchiness scale

- This music is catchy.
- This music is distinctive compared to the other music that I have heard in the experiment so far.
- This music sparked my interest.
- This music is memorable.

Perceived Complexity

- This music sounds complex.

Being Moved scale (not analyzed)

- This music touched me.
- I felt emotionally moved by this music.

Urge to Move scale

- This music evokes the sensation of wanting to move some part of my body.
- I cannot sit still while listening to this music.
- This music is good for dancing.

Pleasure scale

- I like listening to this music.
- This music makes me feel good.
- Listening to this music gives me pleasure.

1. Genre assignment

Which genre do you consider most appropriate for describing this music?

Multiple choice, facultative

- Country / Western
- Downtempo / Ambient
- EDM / Dance
- Folk / Traditional
- Hard Rock / Metal
- Hip-Hop / Contemporary R’n’B
- Jazz / Blues
- Latin
- Pop / Mainstream
- Punk / Alternative
- Reggae / Dancehall
- Rock ‘n’ Roll / Rock
- Soul / Funk
- Other

1. Personal questions

Age

- In which year were you born?

Residence

- In which country do you currently live?

Gender

- How would you describe your gender?

Style preference

How much do you like the following musical styles?

5-point Likert scales from not at all to very much

- Country / Western
- Downtempo / Ambient
- EDM / Dance
- Folk / Traditional
- Hard Rock / Metal
- Hip-Hop / Contemporary R’n’B
- Jazz / Blues
- Latin
- Pop / Mainstream
- Punk / Alternative
- Reggae / Dancehall
- Rock ‘n’ Roll / Rock
- Soul / Funk

Dance preference

How much do you agree to the following statements?

5-point Likert scales from strongly disagree to strongly agree

- I enjoy dancing to music.
- I want to dance to music frequently.

Expertise

How much do you agree to the following statements?

5-point Likert scales from strongly disagree to strongly agree

- I spend a lot of my free time doing music-related activities.
- I can compare and discuss differences between two performances or versions of the same piece of music.

Slider 1-101 from music listener to professional musician

- How would you describe your engagement with music?
- How many years were you engaged in regular practice of a musical instrument (including voice?)
- What instrument do you play?
