# Supplementary material for: The perceived catchiness of music affects the experience of groove
Source: PLoS One. 2024 May 15;19(5):e0303309. doi: 10.1371/journal.pone.0303309 (PMC11095763; doi:10.1371/journal.pone.0303309)
Supplement: S3 File — Details on the samples used for the stimuli creation. (DOCX) [file pone.0303309.s003.docx]

**S3 File. Stimuli samples.** Details on the samples used for the stimuli creation.

For Drums, we used Toontrack EZDrummer 2 (version 2.1.8) and its sound libraries and presets Modern Basic, Modern Metal, Vintage Basic, Funky Tape (Funkmasters expansion, version 1.5.4), One For Dilla (Hip-Hop! Expansion, version 1.0.0). For Bass, we used AmpleSound libraries Bass P III, Bass J III, and Metal Ray5 III (all in version 3.10) for the sounds J Basic, J default, P Basic, Metal Ray Rock, and additionally the Apple sample 80s Pop Bass. For the Keys, we used the Apple samples Classic Electric Piano, Steinway Grand Piano, Wurlitzer Classic, and Flying Clav. Additionally, we used AmpleSound Guitar SC III (version 3.10) for the SC_Shine guitar samples. We deactivated Auto-Buzz and Fingering Noises for the string samples. The stimuli were exported as 192 kBit/s mp3s.

The bass samples have a relatively long envelope to sound realistic. Their perceptual attack time is therefore always behind the beat when quantized, giving the bass a clearly audible laid-back feel. The envelopes of 20 Bass notes with different characteristics were measured in intensity diagrams with the Lucerne Audio Recording Analyzer LARA (version 2.6.3), which showed a mean attack time of 29ms. Therefore, the bass track was set to have a 30 ms pre-delay. For the (distorted) Guitar sample, we used a 50 ms pre-delay, based on the same procedure.

A selection of the stimuli was presented to professional musicians that play the respective instruments to verify the patterns’ ecological validity, and the samples’ authenticity.
