# Supplementary material for: The perceived catchiness of music affects the experience of groove
Source: PLoS One. 2024 May 15;19(5):e0303309. doi: 10.1371/journal.pone.0303309 (PMC11095763; doi:10.1371/journal.pone.0303309)
Supplement: S4 File — Results of the model selection processes. (DOCX) [file pone.0303309.s004.docx]

**S4 File. Model selection.** Results of the model selection processes.

We performed leave-one-out cross validation to test for the optimal model structure, i.e., whether and which random terms are needed. Afterwards, we scanned the selected model for any superfluous variables and recalculated the model without these.

The results of the cross validation for the respective models can be seen in Tables 1-4. Following the suggestion by Sivula et al. [1], we view only ELPD differences > 4 as important, hence, we did not choose the best performing model for catchiness but the most economic one. As ELPDs are, broadly speaking, a result of weighing model fit against added complexity, and as complexity increases drastically (see the number of parameters *p*), the comparison confirms the great importance of including random slopes in datasets such as ours.

**Table 1.** **Perceived Catchiness and Recognition model.** Catchiness – recognition leave-one-out model comparison results, with the best performing model in terms of ELPD highlighted in grey. ELPD is the models’ theoretical expected log pointwise predictive density, p is the number of model parameters, and se the standard error.

| Model | ∆ elpd | ∆ se | elpd | se elpd | *p* | se *p* |
| --- | --- | --- | --- | --- | --- | --- |
| PartSlope + StimSlope | 0.0 | 0.0 | -7477.7 | 89.5 | 1196.0 | 15.3 |
| PartSlope + StimInt | -17.4 | 7.9 | -7495.1 | 89.5 | 1154.0 | 15.1 |
| PartInt + StimInt | -181.2 | 30.1 | -7658.9 | 87.6 | 785.5 | 11.0 |
| PartInt | -245.0 | 32.0 | -7722.7 | 88.7 | 678.4 | 10.7 |
| Fixed only | -2544.5 | 81.0 | -10022.2 | 63.4 | 3.7 | 0.1 |

**Table 2.** **Perceived Catchiness model.** Catchiness leave-one-out model comparison results, with the best performing model in terms of ELPD highlighted in grey. ELPD is the models’ theoretical expected log pointwise predictive density, p is the number of model parameters, and se the standard error.

| Model | ∆ elpd | ∆ se | elpd | se elpd | *p* | se *p* |
| --- | --- | --- | --- | --- | --- | --- |
| PartSlope + StimInt | 0.0 | 0.0 | -4818.5 | 81.9 | 1939.8 | 23.5 |
| PartSlope | -0.8 | 1.7 | -4819.3 | 81.9 | 1925.0 | 23.5 |
| PartSlope + StimSlope | -3.2 | 8.0 | -4821.7 | 81.7 | 2176.8 | 25.6 |
| PartInt | -1000.5 | 56.9 | -5819.0 | 80.3 | 458.7 | 5.5 |
| PartInt + StimInt | -1000.8 | 56.9 | -5819.2 | 80.3 | 476.0 | 5.6 |
| Fixed only | -1636.7 | 67.5 | -6455.2 | 75.5 | 16.0 | 0.2 |

**Table 3**. **Urge to Move model.** Urge to Move leave-one-out model comparison results, with the best performing model in terms of ELPD highlighted in grey. ELPD is the models’ theoretical expected log pointwise predictive density, p is the number of model parameters, and se the standard error.

| Model | ∆ elpd | ∆ se | elpd | se elpd | *p* | se *p* |
| --- | --- | --- | --- | --- | --- | --- |
| PartSlope + StimSlope | 0.0 | 0.0 | -5888.1 | 83.5 | 2359.7 | 25.9 |
| PartSlope + StimInt | -48.8 | 14.8 | -5936.9 | 83.6 | 2024.1 | 23.2 |
| PartInt + StimInt | -677.9 | 47.8 | -6566.0 | 78.6 | 607.8 | 6.9 |
| PartInt | -774.3 | 49.2 | -6662.4 | 79.2 | 474.1 | 5.9 |
| Fixed only | -1428.0 | 61.9 | -7316.2 | 75.2 | 16.0 | 0.2 |

**Table 4.**  **Pleasure model.** Pleasure leave-one-out model comparison results, with the best performing model in terms of ELPD highlighted in grey. ELPD is the models’ theoretical expected log pointwise predictive density, p is the number of model parameters, and se the standard error.

| Model | ∆ elpd | ∆ se | elpd | se elpd | *p* | se *p* |
| --- | --- | --- | --- | --- | --- | --- |
| PartSlope + StimSlope | 0.0 | 0.0 | -5827.2 | 83.0 | 2200.6 | 24.2 |
| PartSlope + StimInt | -28.1 | 12.4 | -5855.3 | 83.2 | 1924.2 | 22.0 |
| PartInt + StimInt | -512.9 | 41.6 | -6340.1 | 79.3 | 531.1 | 5.8 |
| PartInt | -542.7 | 42.0 | -6369.8 | 79.5 | 435.2 | 5.0 |
| Fixed only | -1044.3 | 55.6 | -6871.5 | 77.2 | 15.7 | 0.2 |

1. Sivula T, Magnusson M, Matamoros AA, Vehtari A. Uncertainty in Bayesian Leave-One-Out Cross-Validation Based Model Comparison [preprint]. arXiv; 2022 [cited 2023 Oct 11]. Available from: <http://arxiv.org/abs/2008.10296>
